# Progress on Improved Fouling Resistance-Nanofibrous Membrane for Membrane Distillation: A Mini-Review

**DOI:** 10.3390/membranes13080727

**Published:** 2023-08-11

**Authors:** Yong Zen Tan, Nur Hashimah Alias, Mohd Haiqal Abd Aziz, Juhana Jaafar, Faten Ermala Che Othman, Jia Wei Chew

**Affiliations:** 1 School of Chemistry, Chemical and Biotechnology Engineering, Nanyang Technological University, Singapore 637459, Singapore; ytan075@e.ntu.edu.sg; 2 Department of Oil and Gas Engineering, School of Chemical Engineering, College of Engineering, Universiti Teknologi MARA, Shah Alam 40450, Selangor, Malaysia; 3 Department of Chemical Engineering Technology, Faculty of Engineering Technology, Universiti Tun Hussein Onn Malaysia, Pagoh Higher Education Hub Muar, Batu Pahat 84600, Johor, Malaysia; 4 Advanced Membrane Technology Research Center (AMTEC), School of Chemical and Energy Engineering, Faculty of Engineering, Universiti Teknologi Malaysia, Skudai 81310, Johor, Malaysia; juhana@petroleum.utm.my; 5 Digital Manufacturing & Design Center (DManD), Singapore University of Technology & Design, 8 Somapah Road, Singapore 487372, Singapore; fatenermala.othman@gmail.com; 6 Singapore Membrane Technology Center, Nanyang Technological University, Singapore 637141, Singapore

**Keywords:** membrane distillation, membrane, nanofiber, fouling, modification

## Abstract

Nanofibrous membranes for membrane distillation (MD) have demonstrated promising results in treating various water and wastewater streams. Significant progress has been made in recent decades because of the development of sophisticated membrane materials, such as superhydrophobic, omniphobic and Janus membranes. However, fouling and wetting remain crucial issues for long-term operation. This mini-review summarizes ideas as well as their limitations in understanding the fouling in membrane distillation, comprising organic, inorganic and biofouling. This review also provides progress in developing antifouling nanofibrous membranes for membrane distillation and ongoing modifications on nanofiber membranes for improved membrane distillation performance. Lastly, challenges and future ways to develop antifouling nanofiber membranes for MD application have been systematically elaborated. The present mini-review will interest scientists and engineers searching for the progress in MD development and its solutions to the MD fouling issues.

## 1. Overview of Fouling in Membrane Distillation

Membrane distillation (MD) has been recognized as one of the seawater desalination technologies capable of addressing water-energy constraint issues [1]. In MD applications, the hydrophobic membrane acts as a barrier, preventing feed liquid from directly entering membrane pores while allowing vapor transfer. MD is not limited by the high salt concentration and is suitable for the treatment of high-salinity wastewater [2]. However, commercial hydrophobic membranes are mostly made of low-surface-energy polymers such as polyvinylidene fluoride (PVDF), polypropylene (PP), and polytetrafluoroethylene (PTFE), which cannot avoid pore fouling, especially for the treatment of challenging wastewaters [3,4]. The accumulation of unwanted deposits on a membrane’s surface or inside a membrane’s pores has been one of the challenging issues that need to be overcome for long-term MD operations. It is discovered that fouling in the MD process is still understudied and poorly understood when compared to fouling in the pressure-driven membrane process. However, the MD process also exhibits all identified types of fouling that can occur in a membrane-based process. Fouling agents gradually block the microchannels of the membrane, which leads to less vapor diffusion [5]. The fouling layer introduces additional mass and thermal resistance, which leads to the decline of the driving force required to transport water vapor across the hydrophobic pores of a membrane [6].

The formation of fouling on the membrane surface affects the mass transfer across the membrane, resulting in a decrease in permeate flux. There are three types of mechanisms that explain how gases and vapor are transported through porous media. However, in direct contact membrane distillation (DCMD), the Knudsen flow and molecular diffusion models are most related to the process [7]. When a fouling deposit forms on a membrane’s surface, the mass transfer coefficient experiences an increase in hydraulic and thermal resistance. The degree of resistance is computed based on the characteristics of the contamination film, such as the layer’s thickness and porosity. The fouling layer reduces the difference in temperature across the membrane, resulting in temperature polarization. Temperature gradients across the membrane are recognized as the driving force for flux production. On the other hand, flux decline in MD is due to the blockage of the pore by the fouling layer, which refers to hydraulic resistance [8]. In addition to causing flux decline, fouling frequently causes wetting which subsequently results in the contamination of the permeate by the feed. Additional energy consumption and membrane damage are major consequences in membrane-based operations caused by membrane fouling.

**Figure 1:**
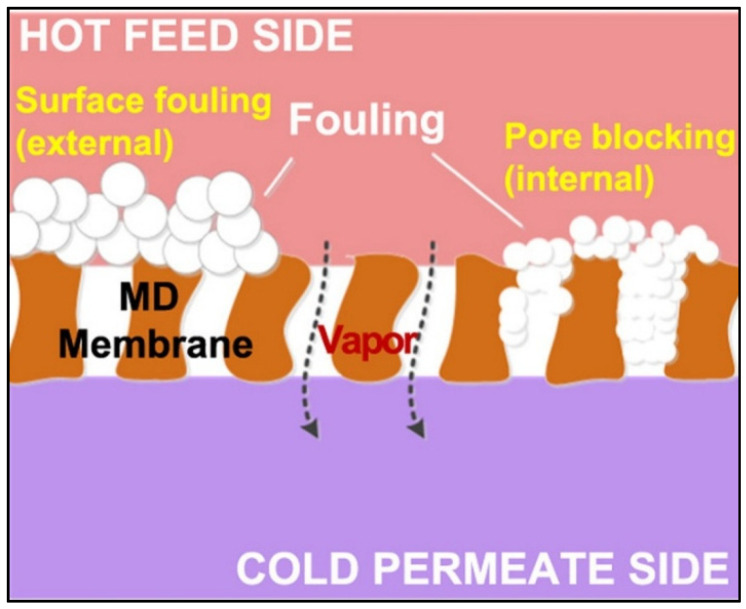
Fouling and its effect on membrane distillation. Reprinted/adapted with permission from Ref. [8].

Fouling in MD in treating wastewater can be broadly categorized into the following three groups: (a) organic fouling, (b) inorganic fouling, and (c) biological fouling. Organic fouling is the result of the deposition of organic compounds such as oil [9], surfactant [10] and textile dyes [11]. Inorganic fouling is mainly due to the deposition of inorganic particulates or hard mineral salts containing chemical species such as calcium, magnesium, sulfate, chloride, carbonate, sodium, silica or iron [12]. Meanwhile, biological fouling results from the build-up of microorganisms such as bacteria [13]. Figure 1 shows the illustration of fouling and its effect on membrane distillation [8].

### 1.1. Organic Fouling

Organic contaminants, primarily produced by industrial effluents, have long been a significant challenge for direct membrane distillation applications. Amphiphilic organic contaminants (e.g., surfactants) and low-surface-tension contaminants (e.g., oils, dyes, and surface-active agents) found in wastewater can significantly reduce the liquid entry pressure of hydrophobic membranes, allowing the pores of hydrophobic membranes to be easily wetted [10,14]. Saline wastewaters (e.g., shale-gas wastewater, electroplating wastewater, dyeing waste) [3] and oily wastewater (e.g., oil and gas exploitation, food, metal processing, textile and leather industries, and domestic sewage) [15] from various sources are examples of challenging wastewaters containing various organic substances that can hardly be treated via large-scale industrial implementation of membrane distillation. On the other hand, the fouling layer could be also caused by the condensation of organic fouling and inorganic fouling containing Ca, Mg and P, originated from elements (C, O, P, Mg, Ca) [16].

**Figure 2:**
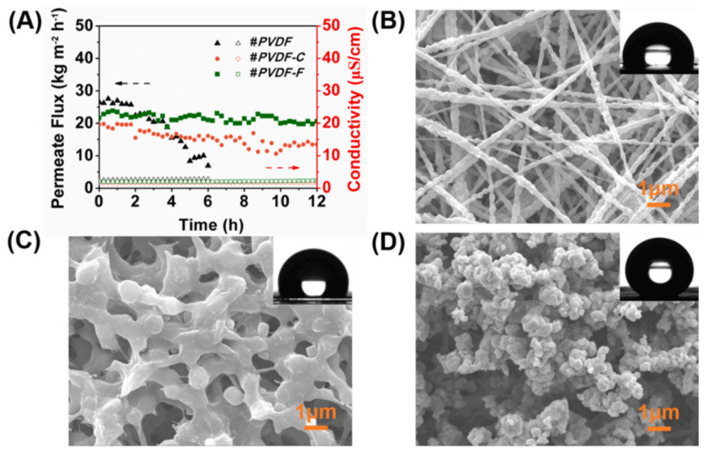
The DCMD performance using a 3.5 wt% NaCl feed solution containing 150 ppm dodecyl trimethyl ammonium bromide (DTAB)-stabilized mineral oil–in-water emulsion. (**A**) Water vapor fluxes and permeate conductivities of nanofibrous PVDF membrane (#PVDF), commercial PVDF membrane (#PVDF−C) and fluorinated re-entrant structure on a dopamine-coated PVDF nanofibrous membrane (#PVDF−F); black arrow (permeate flux) and red arrow (conductivity); SEM and CA images of (**B**) #PVDF, (**C**) #PVDF−C and (**D**) #PVDF−F after MD test. Reprinted/adapted with permission from Ref. [19].

For hydrophobic membranes, surfactant pore wetting is unavoidable. Unfortunately, surfactants can be found in complex wastewater, such as saline wastewater [17,18] and oily wastewater [19]. In addition to lowering feed surface tension, a surfactant can be easily adsorbed onto the hydrophobic membrane surface or even into membrane pores, gradually hydrophilizing the hydrophobic pores [3]. For commercial PVDF and PTFE hydrophobic membranes, it was observed that pore wetting could occur even at the lowest SDS concentration of 0.1 mM, where permeate fluxes began to increase. At the same time, salt rejection decreased drastically [20]. The hydrophilization process of the pores will continue to develop in the depth of the membrane pore channel as the MD operation duration progresses, eventually advancing from partial pore wetting to complete pore wetting. The practical application of the MD process is no longer practicable in this case because the wetted pores provide channels for the permeation of feed wastewater. The micelle formation phenomenon, in which surfactant molecules aggregate and form micelles [21], should also be considered during the MD process due to its impact on surface tension reduction. Micelles form when the surfactant concentration exceeds the critical micelle concentration (CMC) [21]. When the surfactant concentration at the hydrophobic membrane/water interface exceeds the CMC, the surfactant monomers can be distributed predictably, with the hydrophobic tails towards the hydrophobic membrane’s surface and the hydrophilic heads towards the aqueous solution, resulting in the hydrophobic membrane no longer acting as a barrier to liquid water permeation [4]. For instance, this phenomenon was observed on hydrophobic electrospun nanofibrous PVDF and commercial PVDF membranes when the membranes were tested with complex feed components containing surfactants and oil [19]. As shown in Figure 2, both the electrospun nanofibrous PVDF membrane and the commercial PVDF membrane experienced decreased water flux. Within 6 h, the flux of the electrospun nanofibrous membrane had dropped dramatically. During a 12 h MD operation, the flux of commercial PVDF membrane maintained a continuous downward trend. On the other hand, the omniphobic fluorinated re-entrant structure on a dopamine-coated PVDF nanofibrous membrane demonstrated consistent MD performance with a stable water flux.

**Figure 3:**
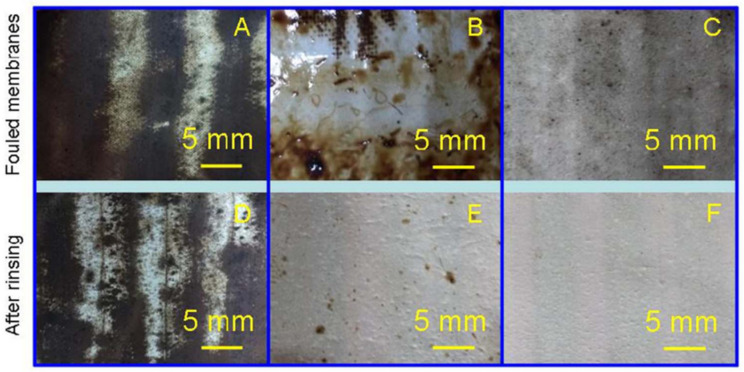
The photographic images of the membranes after membrane distillation and the membranes after rinsing 2 min with deionized water (**A**,**D**) the commercial PTFE membranes, (**B**,**E**) the PTFE/CA-nanofibrous composite membranes, and (**C**,**F**) the PTFE/CA-SiNPs-nanofibrous composite membrane. Reprinted/adapted with permission from Ref. [26].

Oil is one of the most significant pollutants in wastewater due to its persistence and toxicity [22,23,24], and its presence can harm aquatic ecosystems by increasing chemical oxygen demand levels [25]. MD has been explored for oily wastewater treatment using hydrophobic membranes; however, due to long-range hydrophobic-hydrophobic interactions, the hydrophobic membranes employed in MD are easily fouled by oil, resulting in wetting and, consequently, a loss of selectivity of the membranes. For example, PTFE substrate and PTFE substrate coated with cellulose acetate fibrous network exhibited severe membrane fouling during the MD process with saline feed containing 600 mM NaCl and 1000 mg/L crude oil [26]. After the MD process, the oil fouling can be visible on the PTFE substrate and PTFE substrate/cellulose acetate fibrous membrane, as shown from a photographic image of the membrane surface (Figure 3A,B). Severe wetting and oil fouling on the membrane surface were also observed on a hydrophobic copolymer vinylidene fluoride–hexafluoropropylene (PVDF-HFP) nanofibrous membrane, with a sharp reduction in salt rejection and an increase in water flux when the oil concentration reached 80 mg/L in saline water [27]. Hou et al. [28] investigated the interaction between an oil droplet and an electrospun composite membrane surface using oil–probe force spectroscopy better to understand a membrane’s fouling propensity in oily foulants. The analysis provides a complete interaction curve that includes two stages: advancing (approach, contact, and compassion) and receding (retract, split and detach). The membrane has extremely low oil adhesion if the maximum adhesive force in the corresponding receding curve is the same as the baseline shift [27]. However, this is not true for most hydrophobic membranes previously reported for MD studies [29].

Previously Laqbaqbi et al. [30] studied filtration for cationic and anionic dyes using membrane distillation. They reported that the membrane fouling occurred due to the interaction between dye molecules and membrane surface and thus influenced vapor flux across the membrane. This interaction caused the dye contaminants to deposit on the membrane surface and within membrane pores. Further, as most hydrophobic polymeric membranes reported in the literature have a negatively charged surface, positively charged dye molecules (e.g., methylene blue and crystal violet) are more likely to foul the membrane [30,31,32]. For instance, these cationic dyes adsorbed easily onto the membrane surface due to the electrostatic attraction in the case of methylene blue or the hydrogen bonding between the negative functional group of the membrane and the electron-donating nitrogen atoms in the case of crystal violet [32]. Negatively charged dye molecules (e.g., Congo red and sodium fluorescein), on the other hand, as compared to cationic dyes, generated repulsive forces with the membrane surface, resulting in the creation of a dye–dye structure on the membrane surface (i.e., cake formation) and less influence on pore size reduction [30,31].

Natural organic matter (NOM) is the most common organic fouling developed in membrane-based technologies [8]. It is present in every natural water resource [33]. The presence of natural organic matter is still a highly problematic issue regarding MD technologies [34]. Humic acid, a complex mixture of organic acids with phenolate and carboxyl groups, is the principal constituent of NOM foulants [8]. It is known to be favorably adsorbed on hydrophobic surfaces [35]. Humic acid is formed through the microbial degradation of low to moderate molecular weight of organic matter, giving the water a characteristic yellowish-brown color [36]. It is typically present in the range of 2–5 mg/L in seawater [37,38]. Its surface tension, for a concentration of 1000 mg/L, has been reported to be around 59 N/m [39], which is lower than the surface tension of water (72 N/m). NOM can be adsorbed onto a membrane’s surface through different mechanisms, such as specific chemical affinity and electrostatic and hydrophobic interactions. Higher organic compound adhesion on the membrane has been observed when a higher temperature was used during operation [40]. At higher temperatures, the disaggregation of humic substances occurs, which creates smaller molecular-sized humic that result in higher membrane fouling rates. Once fouled, cleaning the deposited organic compounds without using chemicals is usually not easy. It has been found that humic acid fouling can be thoroughly cleaned using basic solutions of 0.1 M NaOH to achieve high initial flux recovery [41]. Despite external fouling control, the process requires extra chemical consumption, making it cost more operationally and less environmentally friendly.

### 1.2. Inorganic Fouling

**Figure 4:**
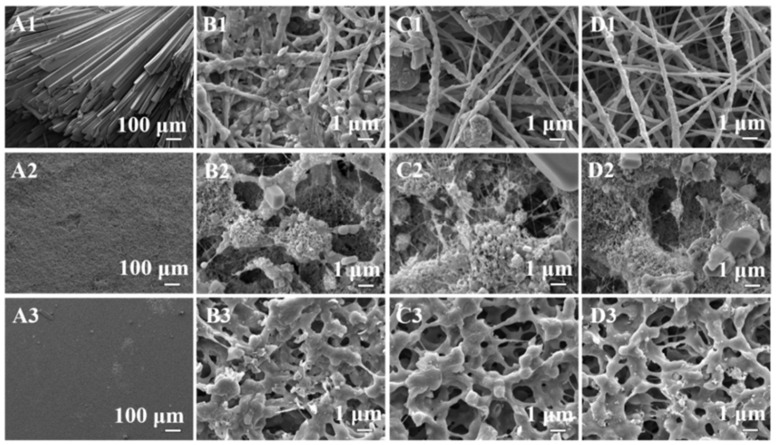
SEM images of the surfaces of (1) nanofibrous PVDF, (2) superhydrophobic/nanofibrous PVDF and (3) commercial PVDF membranes after DCMD testing using the feed solutions containing CaSO_4_ (**A1**–**A3**), HA (**B1**–**B3**), TDAB (**C1**–**C3**) and SDS (**D1**–**D3**), respectively. Reprinted/adapted with permission from Ref. [43].

Inorganic fouling generally refers to depositing precipitated hard minerals during membrane distillation. Inorganic scaling, such as sodium chloride, barium sulfate, calcite, gypsum, silica, strontium carbonate, calcium carbonate and calcium sulfate, are widely found to be the main culprit of membrane scaling in MD desalination. These scale-forming species were also observed in saline wastewater. The buildup of scales on the membrane’s surface or inside the membrane pores obstructs water vapor’s mass movement across the membrane, eventually rendering the membrane hydrophilic, which can lead to membrane wetting [14]. Both of these phenomena can harm membrane permeate flux and salt rejection. Commercial hydrophobic polymers for electrospun nanofibrous membrane fabrication have insufficient hydrophobicity, resulting in membrane scaling and failure in long-term operation [42,43]. For example, after a 20 h operation, the permeation flux of the nanofibrous PVDF membrane decreased, accompanied by a significant increase in conductivity at the permeate side due to CaSO_4_ crystals aggregation on the membrane’s surface. On the other hand, a membrane with higher hydrophobicity comprised of a superhydrophobic surface layer composite with underlying electrospun nanofibrous PVDF support demonstrated consistent performance. A comparison fouling study with different membrane (e.g., nanofibrous PVDF, superhydrophobic/nanofibrous PVDF and commercial PVDF membranes) and different foulants (e.g., CaSO_4_, humic acid (HA), N, N, N-trimethyl-1-dodecanaminium bromide (TDAB), and sodium dodecylsulfonate (SDS) was shown in Figure 4 [43].

**Figure 5:**
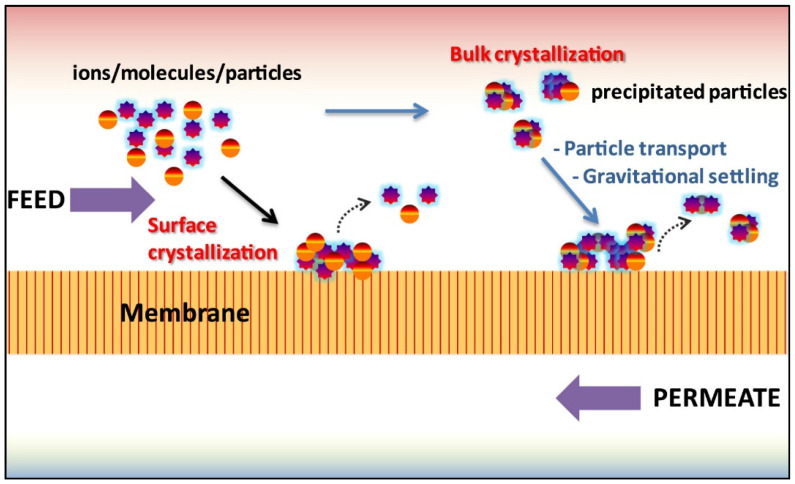
An illustration of the surface (heterogeneous) and bulk (homogeneous) crystallization mechanisms during inorganic fouling of membrane distillation. Reprinted/adapted with permission from Ref. [8].

In MD processes, the rate of scale formation can be governed by several factors, including the degree of supersaturation, temperature, water composition, flow conditions, the material of the substrate, and the availability of any nucleation sites. Generally, scaling involves two mechanisms which are surface crystallization and bulk crystallization. The former is called heterogeneous crystallization; the latter is termed homogeneous crystallization, and both nucleations exist on the membrane surface [8,44]. Homogeneous nucleation occurs in a saturated solution, while heterogeneous nucleation in an unsaturated solution requires less energy [45]. As a result, heterogeneous nucleation is more likely, to contribute to membrane surface scaling [46]. The illustration of these two mechanisms during inorganic fouling in MD is shown in Figure 5. The relation between those mechanisms can be understood by the Gibbs free energy for homogeneous (Δ*G*_homogeneous_) and heterogeneous (Δ*G*_heterogenous_) nucleation as shown in Equation (1):

(1) $$\frac{\Delta G_{heterogenous}}{\Delta G_{homogenous}}=\frac{1}{4}\;\left(2+cos\theta \right)){\left(1-cos\theta \right)}^{2}{\left(1-E\frac{{\left(1+cos\theta \right)}^{2}}{{\left(1-cos\theta \right)}^{2}}\right)}^{3}\;$$

where θ is the membrane-crystal-liquid static contact angle, and ε is the membrane surface porosity. Δ*G*_heterogenous_ or Δ*G*_homogeneous_ is the energy barrier for heterogeneous nucleation or homogenous nucleation process induced by a porous membrane. For instance, the higher the value of Δ*G*_heterogenous_, the more difficult it is for salt crystals to overcome this energy barrier and generate heterogeneous nucleation on the membrane [47]. In general, to reduce membrane scaling tendencies, the heterogeneous nucleation barrier can be improved by lowering the contact area and shortening the contact duration between fluid and membrane surface, which is attainable with a membrane with a larger contact angle and porosity [44]. Park et al. [45] observed that fluorine-containing thermally rearranged nanofiber membranes had high heterogeneous nucleation energy, causing crystal nucleation on the membrane surface to be disrupted. This is because fluorine atoms are non-polar and have low interaction energy with water molecules, which helps enhance the hydrophobicity of the prepared membrane. For these reasons, fluorine-containing rearranged nanofiber membranes thermally outperform non-fluorine-containing thermally rearranged nanofiber membranes regarding membrane distillation performance across the full feed temperature range.

### 1.3. Biofouling

Biofouling refers to the undesirable accumulation of waterborne microorganisms, which may result from the deposition and growth of bacteria cells on the surface or inside the membrane [48]. Biofouling has been problematic in many membrane-based filtration technologies because bacteria are naturally present in many water sources [49]. It is more predominant in food, dairy farms, and industrial waste industries where harsh feed solutions are treated [50]. However, in practical MD applications during which the feeds are processed at high temperatures and have high salinity, microbial growth is restricted to a great extent [51]. Biofouling may have caused significant drawbacks to other membrane technologies, such as reverse osmosis (RO), nanofiltration (NF), and microfiltration (MF), but not much for MD.

## 2. Development of Antifouling Nanofibrous Membranes for Membrane Distillation

Membrane distillation membranes with special wettability, such as superhydrophobic membranes, omniphobic membranes, or asymmetric-wettability-surface membranes (Janus), have garnered a lot of attention in recent years as a way to improve antifouling and antiwetting properties. The acting force between solid and liquid phases is well understood to be highly dependent on the composite interface’s chemical composition and surface roughness. For antifouling and antiwetting membranes, a surface with high Cassie–Baxter state stability is desirable. This is because the surface is not favorable to liquid penetration in the Cassie–Baxter state, as air pockets are trapped between the liquid droplet and the membrane surface due to surface roughness. This condition is required to create surfaces with low adhesion and strong liquid repellency, allowing a droplet to roll off a substrate readily. To attain a membrane with a stable Cassie–Baxter state, surface roughness and surface chemistry should be considered. This state can be described as follows:

(2) $$Cos\theta_{CB}=f_{sl}\;Cos\theta_{e}-f_{lv}$$

where *f*_sl_ is the area fraction of the solid–liquid interface, and *f_lv_* is the area fraction of the liquid–air interface underneath the liquid droplet. The summation of both *f*_sl_ and *f_lv_* is equal to one. According to Equation (2), as *f_lv_* grows, the contact angle of the surface will always increase, implying that as trapped air in cavities increases, the probing liquid will only come into touch with a tiny portion of the surface, resulting in increasing hydrophobicity. This is achievable when the surface has low surface energy with high surface roughness. Low-surface-energy materials can reduce intermolecular forces and adhesive force, and surface roughness, on the other hand, can intensify the hydrophobicity of a low-surface-energy surface [52].

Electrospun nanofibrous membrane has gained popularity in recent years as an approach for fabricating membranes with special wettability due to its commonalities and desirable intrinsic membrane properties such as high porosity and rough hierarchical structures, which are a good criterion for forming the “Cassie–Baxter” state at the solid–liquid interface [18]. Furthermore, the electrospun fibrous network’s high porosity and interconnected open structure can result in high membrane permeability [20]. However, the hydrophobicity of electrospun nanofibrous membrane depends on the polymers used and is insufficient to prevent wetting in long-term operation [53]. As a result, considerable effort has been devoted to making the surface of electrospun nanofibrous membranes more hydrophobic. This can commonly be accomplished by surface modification with low-surface energy chemicals such as fluorosilane and organosilicon. Hydrophobic surfaces can be classified into superhydrophobic, omniphobic and Janus surfaces, which will be discussed in further details in the following sections of this review. Superhydrophobic membranes consist of purely low-surface-energy material on both surfaces which can be combined with micro/nanoscale structures to repel polar liquid. Omniphobic membranes are composed of both high and low-surface-energy materials on both surfaces which repel both polar and non-polar liquid. Janus membranes as its name suggests consist of two different materials on the two different surfaces of the membrane, allowing the two surfaces to be either hydrophilic, hydrophobic or omniphobic independent of each other. Superhydrophobic membranes are known to have higher resistance against scaling induced wetting, while omniphobic membranes are known to have higher resistance against surfactant induced wetting [54,55]. However, with Janus membranes, membranes can be designed to have two different surfaces to make the membrane more robust against scaling and surfactant induced wetting. Furthermore, all of these surfaces can be modified with bactericidal metal or metal nanoparticles or polymers [56,57], to resolve the issue with biofouling along with inorganic (scaling) and organic (surfactant) fouling.

### 2.1. Superhydrophobic Nanofibrous Membrane

In general, superhydrophobic surfaces exhibit water contact angles above 150°. Despite the fact that some surfaces had a water contact angle greater than 150°, the water droplets stuck strongly to the surfaces that were easily wetted during the MD process [43]. Another requirement for the superhydrophobic surface is that the contact angle hysteresis must be lower than 10° [58]. Contact angle hysteresis is the resistance to droplet movement along a solid surface. Contact angle hysteresis can be calculated by subtracting the advancing contact angle (maximal contact angle) from the receding contact angle (minimal contact angle) [59]. The superhydrophobic surface was inspired by a well-known natural phenomenon known as the “lotus effect” by lotus leaves, which exhibits extreme water-repellency as water droplets easily roll off the surface [60]. This self-cleaning property is due to the low surface energy of hydrophobic epicuticular wax on the surfaces of the leaves, as well as the micro/nanoscale roughness from papillae, both of which are critical in minimizing the contact area between liquid and solid at the interface [61].

**Figure 6:**
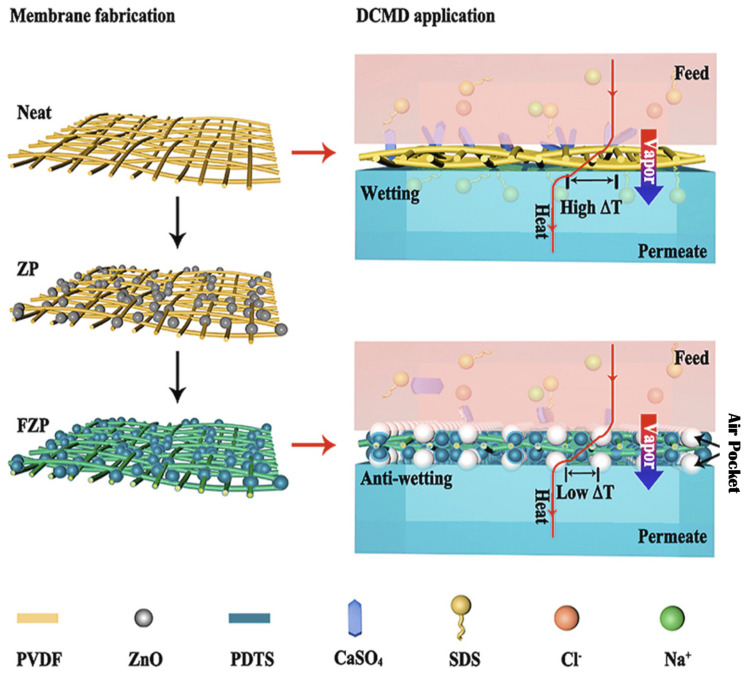
The schematic diagram of the fabricated superhydrophobic nanofibrous membrane fabricated through fluorinating of ZnO blended electrospun PVDF membrane (FZP membrane). Reprinted/adapted with permission from Ref. [47].

A myriad number of methods have been employed to modify hydrophobic electrospun nanofibrous membranes to be superhydrophobic. For instance, Li et al. [47] successfully developed a superhydrophobic nanofibrous membrane with a high water contact angle of 162.3° and sliding contact angle of 9.8° through fluorinating of zinc oxide (ZnO) blended electrospun polyvinylidene fluoride (PVDF) membrane (FZP) as shown in Figure 6. The hierarchical structure of ZnO and the electrospun PVDF membrane, together with low surface energy from 1H, 1H, 2H, and 2H-perfluorodecyl-triethoxysilane, aid in the achievement of superhydrophobicity. When desalinating both pure NaCl solution and NaCl solutions with low-surface-tension sodium dodecyl sulphate (SDS) and sparingly soluble salt calcium sulphate, the FZP membrane has much better anti-wetting properties than neat nanofibrous PVDF membranes due to its superhydrophobicity. Meanwhile, Ren et al. [62] attempted to make PVDF electrospun membranes superhydrophobic by first coating the membrane surface with TiO_2_ nanoparticles using a low-temperature hydrothermal process and then fluorosilanizing the TiO_2_ coated membrane with a low-surface-energy material of 1H, 1H, 2H, 2H-perfluoro-dodecyl trichlorosilane (FTCS). TiO_2_-FTCS modified PVDF electrospun nanofibrous membrane had a higher water contact angle of 157.1°, which increased 12.5% over the corresponding value of virgin PVDF electrospun nanofibrous membrane. Long-term DCMD processes by TiO_2_-FTCS modified PVDF electrospun nanofibrous membranes using actual reverse osmosis brine as feed solution also outperformed commercial PVDF membranes and unmodified PVDF electrospun nanofibrous membranes. However, stable MD performance for superhydrophobic membranes is often limited to short period of time [63,64], far from sufficient to demonstrating stable antiwetting or antiscaling performance of the membranes. Therefore, leading to the development of omniphobic membranes to mitigate these shortcomings of the superhydrophobic membranes.

### 2.2. Omniphobic Nanofibrous Membrane

**Figure 7:**
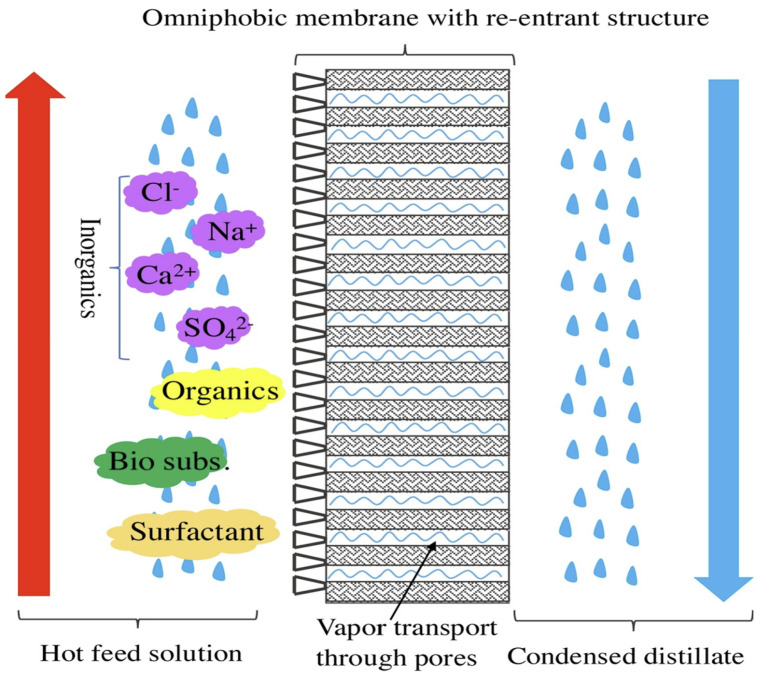
Schematic diagram of membrane distillation by an omniphobic membrane with condensed distillate of water (blue colour). Reprinted/adapted with permission from Ref. [66].

Omniphobic surfaces are more promising because they repel high (water) and low surface tension liquids (alcohol, oil, etc.). In contrast, superhydrophobic membranes do not allow for low surface tension liquids. Aside from chemical composition and surface roughness, re-entrant structure, or surface topography with overhanging features, should be considered an important design factor for surfaces more resistant to low surface tension liquids [53,65]. Figure 7 shows the general schematic diagram of membrane distillation by an omniphobic membrane [66]. Nanofibrous membranes provide an ideal substrate for omniphobic membranes since the bottom-half of the electrospun fibrous network constitutes a re-entrant structure [67]. Spherical cavities, overturned truncated cones, and pillars with side facets are other examples of geometry with re-entrant structure [68]. The re-entrants structure can also prevent foulant accumulation on the surface because the contact area at the liquid–solid interface is reduced [69,70]. The complementary roles of the microstructure and nanostructure can further increase the stability of omniphobic surfaces. This is because air is trapped at multiple length scales for a hierarchically textured surface and, therefore, can support a stable Cassie–Baxter state compared to the trapped air for a single-tiered textured surface.

The application of omniphobic membranes for membrane distillation was first conducted by Lin et al. [71] by using inorganic nanofibrous membranes. A five-step approach was employed by the group to develop the re-entrant structure and to reduce the surface energy of the glass fiber membrane through the deposition of silicon dioxide (SiO_2_) nanoparticles that were followed by fluorination and polymer coating with poly(vinylidene fluoride-co-hexafluoropropylene (PVDF-HFP). The membrane displayed omniphobic properties and was very robust, even in the presence of a surfactant. As a comparison, a hydrophobic PTFE could easily be wetted by the surfactant during a DCMD process. Hou et al. [20] developed an omniphobic membrane for anti-surfactant-wetting membrane distillation (MD) by electrospinning hybrid nanofibers made of cellulose acetate and silica nanoparticles to create hierarchical re-entrant surface structures, followed by surface fluorination to lower the fibrous membrane’s surface energy. During the DCMD experiments, the omniphobic membrane outperformed the hydrophobic membranes’ wetting resistance to the low-surface tension feed. It maintained stable permeate flux and salt rejection, whereas surfactants easily wetted the tested hydrophobic membranes. However, according to Feng et al. [54], the omniphobic membrane is prone to fouling during treatment of oily water due to the strong underwater hydrophobic attraction between the membrane surface and the oil droplets and they suggested Janus membrane can substantially mitigate this problem because of lack of attraction between the mineral oil droplets and the hydrophilic membrane surface.

### 2.3. Janus Nanofibrous Membrane

While omniphobic membranes consist of homogenous surface material and surface composition, Janus membranes have asymmetrical surface wettability and frequently incorporate hydrophilic/hydrophobic dual-layer architectures. This surface was inspired by the characteristics of marine creatures such as fish scales, clamshells, and sharkskin [29]. Fouling can be reduced by using hydrophilic surfaces; however, MD uses hydrophobic surfaces; thus, incorporating both hydrophilic/hydrophobic surfaces has sparked much interest as a potential candidate for a robust antifouling MD membrane [72]. Janus membranes have a hydrophilic surface layer that strongly adsorbs water molecules via the hydrogen-bond interaction, forming an active hydration layer that gives the membrane underwater oleophobicity [9]. This hydration layer prevents oil droplets from spreading on the membrane surface, preventing pores from blocking the underlying hydrophobic membrane. In most cases, hydrophobic porous substrates were modified through the deposition of hydrophilic materials, such as polyethylene glycol (PEG), 18 polydopamine, hydrogels, inorganic/composite and electrospun hydrophilic polymers, or through creating hydrophilic functional groups [73]. When using a Janus membrane in the MD process, the feed solution comes into direct contact with the hydrophilic layer of the membrane rather than the hydrophobic base membrane [74].

**Figure 8:**
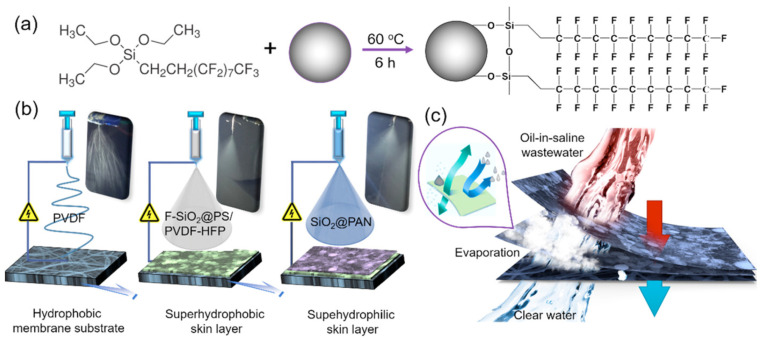
Schematic representation of the (**a**) fabrication of superhydrophobic SiO_2_ NPs. (**b**) fabrication of the Janus skin layer with asymmetric superwettability on a necklace-structured PVDF NFM. (**c**) the proposed mechanism of the antifouling and antiwetting properties using an asymmetrically superwettable Janus skin layer and a plausible mechanism of vapor (red arrow) permeation across the porous membrane with clean water (blue arrow). Reprinted/adapted with permission from Ref. [29].

For instance, Zhu et al. [29] created a Janus membrane by sequentially electrospinning and electrospraying an asymmetrically superwettable Janus skin and a hydrophobic nanofibrous membrane. The electrosprayed asymmetrically superwettable Janus skin comprised nano/microstructured nanofilaments and demonstrated an intriguing underwater superoleophobicity of 164° and an in-air superhydrophobicity of 166°. The fabricated membrane demonstrated high resistance to membrane fouling while maintaining a high flux. Zhu et al. [75] developed a monolithic and self-roughened Janus fibrous membrane with asymmetrical superwettability via sequential electrospinning and electrospraying in combination with thermal treatment. The upper layer of Janus shows underwater superoleophobicity. Once the oil droplets in the hypersaline wastewater encountered the superhydrophilic skin layer, the oil droplets would be effectively rejected because the high-surface-energy water in the superhydrophilic membrane pores was challenging to be replaced by the low-surface energy oil droplets. Figure 8 shows example of the schematic representation of the (a) fabrication of superhydrophobic SiO_2_ NPs (b) fabrication of the Janus skin layer with asymmetric superwettability on a necklace-structured PVDF NFM, (c) the proposed mechanism of the antifouling and antiwetting properties using an asymmetrically superwettable Janus skin layer and a plausible mechanism of vapor permeation across the porous membrane [29].

Overall, based on literature studies on the performance comparison and mechanistic explanations, none of these MD membranes can resolve most of challenges at the same time especially on fouling, wetting and scaling issues. For instance, even though omniphobic membranes can prevent wetting and scale, they are susceptible to fouling due to hydrophobic interactions in the water. Janus membranes with a dense hydrophilic surface layer can prevent wetting and fouling, but they are vulnerable to scaling due to the precipitation and crystallization of salts within the surface layer. Therefore, further modification on the membranes is essential to ensure the maximize the performance of MD membranes.

## 3. Modifications on Nanofiber Membrane for Membrane Distillation

Ideally, a membrane for membrane distillation (MD) should demonstrate a high liquid entry pressure (LEP), high void volume fraction such as porosity and narrow pore size distribution, low pore tortuosity, high hydrophobicity, low thermal conductivity, antifouling properties, and robust for prolonged operation [29,76]. Electrospun nanofiber membrane was first applied to MD in 2008 [77], and ever since then, various advantages of electrospun nanofiber have been explored as an ideal material for MD membrane design, such as easy-to-attain superhydrophobic surface, adjustable fiber diameter, interconnected structure, thermal stability, high surface or volume ratio and porosity.

**Figure 9:**
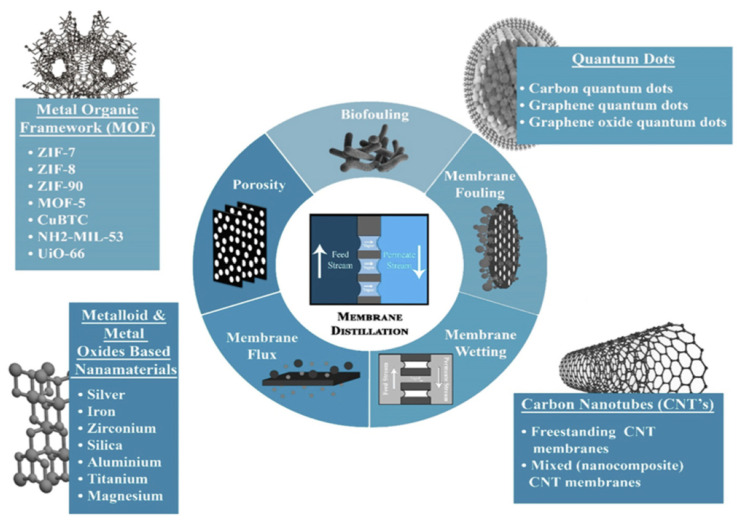
Recent evolution in MD membranes with enhancement in several major areas. Reprinted/adapted with permission from Ref. [80].

However, pristine nanofiber membranes have been reported to suffer from membrane wetting due to a lack of hydrophobicity, big pore sizes, and fewer surface functionalities [78]. Thus, many groups have attempted to improve the pristine nanofiber properties, and membrane modification for MD application is a recent issue and has only been studied since 2011 [79]. In addition, immense research has also been devoted to enhancing further the nanofiber membranes’ performance by emphasizing improvements in the material and structures of the membranes. Figure 9 shows the evolution of MD membranes targeting enhancement in major areas such as membrane fouling, biofouling, membrane wetting, membrane flux and porosity properties as a new generation of nanofiber membranes for efficient MD output.

Other than that, consideration should also be given to the desired properties of the membranes that also contemplate their interdependencies [79]. For example, high membrane porosity could facilitate high permeate fluxes, but it could somehow cause the membrane to cope with the issue of wetting and fouling. In addition, the increase in liquid entry pressure (LEP) is associated with the decrease in the membrane pore size, thus consequently decreasing the permeate flux. Therefore, enlightening strategies should be adopted to consolidate the efforts and ensure an effective MD operation.

**Figure 10:**
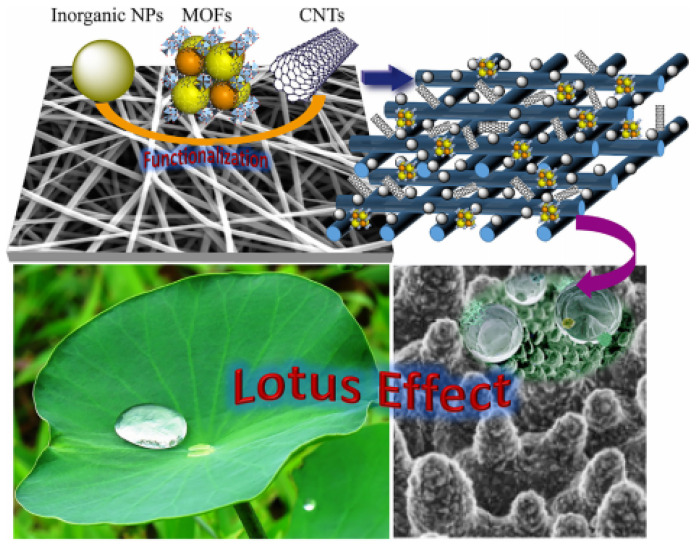
Nanomaterials functionalization on ENMs to construct superhydrophobic lotus effect to enhance the DCMD performance. Reprinted/adapted with permission from Ref. [82].

Altering the surface-wetting properties of these MD membranes by incorporating the membrane with nanomaterials has shown remarkable achievement in developing anti-wetting surfaces of MD membranes. Roughness and surface chemistry are two vital criteria to determine the wettability capability of the nanofiber membranes. According to Pan et al. [81], Wenzel and Cassie’s models reveal that micro/nanoscale surface roughness or surface energy can represent the membranes’ hydrophobicity/hydrophilicity properties. On top of that, extensive efforts have been made to boost the water resistance of the membrane surface by using nanomaterials via increasing surface roughness and forming numerous air pockets. These low dimensional nanomaterials demonstrate favorable properties for MD applications such as high specific surface area, high strength, tuneable hydrophobicity, enhanced vapor transport, high thermal and electrical conductivity and others. Up to date, among the nanomaterial groups selected to be functionalized onto or into MD nanofiber membranes are carbon-based materials, metal–organic frameworks (MOF), and metallic and metal oxides-based nanoparticles (NPs), as shown in Figure 10.

### 3.1. Carbon-Based Materials

**Figure 11:**
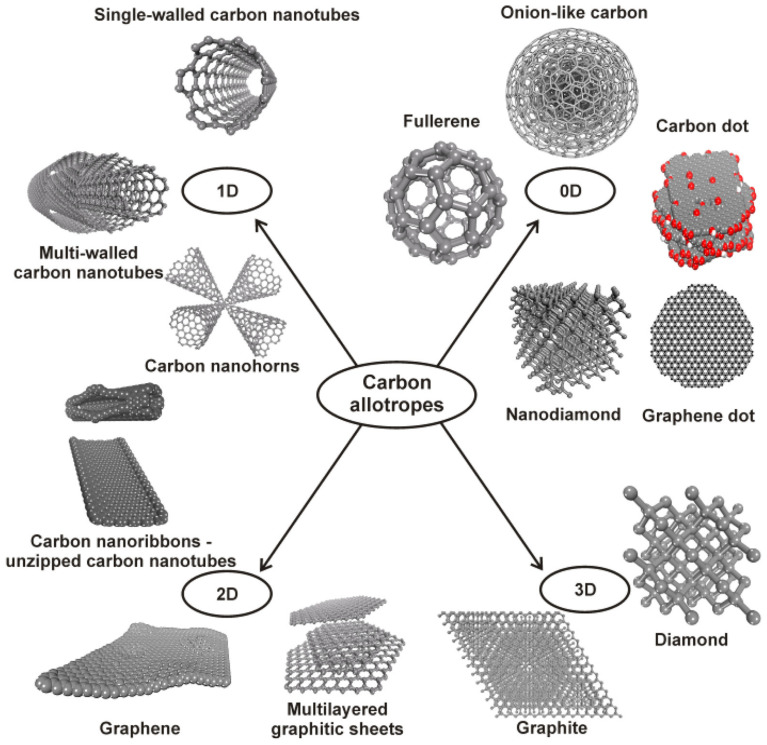
Potential carbon-based materials with different forms of carbon allotropes for MD applications. Red color indicates carbon dot presence 0D carbon allotropes. Reprinted/adapted with permission from Ref. [84].

Carbon has been one of the chief elements in the earth’s civilization, forming stronger bonds than any other materials in different forms [83]. Carbon-based nanomaterials such as carbon nanotubes (CNTs), graphene, and graphene oxide are superior materials that are extensively utilized in membrane separation techniques as they allow water molecules to be transported swiftly and impart an antifouling character to the membrane, making them a successful candidate for MD technology. Figure 11 illustrates the carbon-based materials with different forms of carbon allotropes that have high potential in MD applications [84].

As the electrospun nanofiber membranes (ENMs) suffer much from wetting issues during the long-term operation of MD, Yan et al. [53] fabricated superhydrophobic polyvinylidene fluoride (PVDF) ENMs incorporated carbon nanotubes (CNTs) via a facile spraying method with heat-pressed application to have good bonding force with CNTs. They reported that the high hydrophobicity and low surface energy of CNTs caused the membranes to exhibit the highest water flux (28.4 kg/m^2^ h) with steady vacuum membrane distillation (VMD) performance of more than 26 h. Upon the addition of CNTs in the membranes, the water contact angle increased from 135.4° to 159.3° with increased CNTs density from 4 g/m^2^ to 30 g/m. Moreover, the morphological observation on CNTs-coated membranes also had no significant change after the VMD test, confirming the stability of the CNTs-coated membrane throughout the VMD operation.

**Figure 12:**
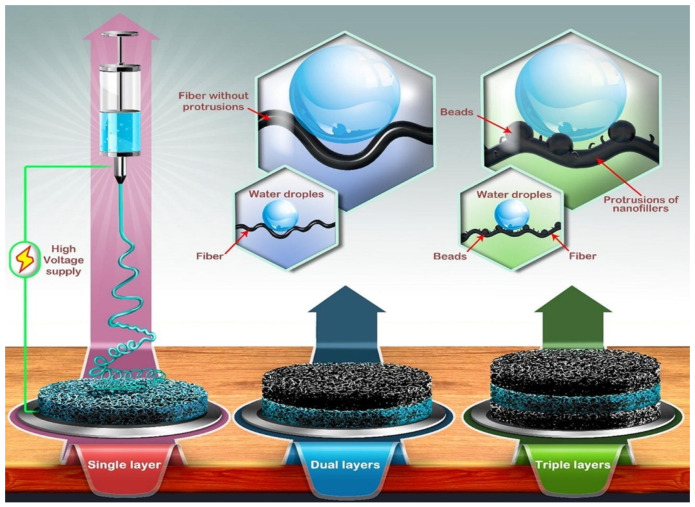
Schematic diagram of the fabricated single, dual, and triple-layered mixed matrix ENMs (MM-ENMs) prepared from multi-walled carbon nanotubes (MWCNs) and graphene oxide (GO) as nanofillers. Reprinted/adapted with permission from Ref. [85].

Recently, Essalhi and co-workers [85] have prepared a robust superhydrophobic mixed matrix electrospun nanofibrous membranes (MM-ENMs) from PVDF-modified multi-walled carbon nanotubes (MWCNs) and graphene oxide (GO) as the nanofillers independently. High loading of MWCNS and GO nanofillers promoted bead formation on the nanofiber membrane. It thus induced smaller inter-fiber space of MM-ENMs prepared, thus resulting in a superhydrophobic character and improvement of the liquid entry pressure of the membranes. They also found out that smaller inter-fiber spaces were obtained with GO nanofiller than with MWCNs, regardless of the choice of the middle layer polymer, due to the extra compaction of the nanofibrous network in the nanofiber membranes and increased agglomeration welding of nanofiber webs when using GO as nanofiller rather than MWCNs. The study concluded that integrating MWCNs into the active surface of dual-layered-MM-ENMs is an attractive strategy for superior DCMD desalination performance. The fabricated triple-layered-MM-ENMs, with an internal hydrophilic layer sandwiched between two hydrophobic layers, exhibited substantial water condensation with water pocket formation, which is not favorable. Within 10 h of MD operation, dual-layered MM-ENM obtained the best ultra-high permeate fluxes of up to 74.7 kg/m^2^ h with electrical conductivity of 7.63 μS/cm and a salt (NaCl) rejection up to 99.995%. Figure 12 shows the schematic diagram of the fabricated single, dual, and triple-layered mixed matrix ENMs (MM-ENMs) prepared from multi-walled carbon nanotubes (MWCNs) and graphene oxide (GO).

In other work performed by Leaper et al. [86], they fabricated reduced graphene oxide functionalized (rGO) with superhydrophobic polyhedral oligomeric silsesquioxane (POSS) molecules (POSS-rGO), showed improved thermal stability and mechanical properties of polymer composites due to strong hydrophobic interactions within the matrix. The flux of the best-performing rGO-enhanced membrane was 21.5% higher than the pristine PVDF membrane and, in fact, almost double the commercial polytetrafluoroethylene (PTFE) membrane after 24 h of testing, with rejection values exceeding 99.9%. Furthermore, the flux of this membrane was stable over 5 days (~28 L m^−2^ h^−1^). Functionalizing GO with POSS and thermally reducing it also allows for porosities exceeding 90%, boosting the hydrophobicity, lowering the chance of pore wetting, and improving the quality of the MD permeate.

Fouladivanda and co-workers also made a similar effort [87], utilizing GO to attain a highly hydrophobic structure, small pore sizes, and a high LEP of MD membrane to guarantee its durability and antipore-wetting properties. A superhydrophobic octadecyl amine-reduced graphene oxide (ODA-rGO)/PVDF-HFP mixed-matrix membrane with a water contact angle of 162° was fabricated. Superhydrophobic additive ODA-rGO was synthesized via the hydrothermal method and further electrospun in PVDF-HFP polymeric dope solution. On top of that, a slight amount of LiCl was also added into the dope prior to electrospinning to compensate low conductivity of ODA-rGO as well as decrease the mean pore size of the electrospun nanofiber from 1.30 to 0.24 µm. The prepared membranes were hot-pressed to improve liquid entry pressure (LEP) and high mechanical strength from 30.4 to 127.6 kPa. With the optimum heat-treated, the fabricated ODA-rGO)/PVDF-HFP mixed-matrix membranes showed impressive performance of air gap membrane distillation (AGMD) test where it obtained an average flux of 21.1 kg/m^2^ in 4 days with the salt rejection of 99.99% along, without any discernible drop in its salt rejection.

**Figure 13:**
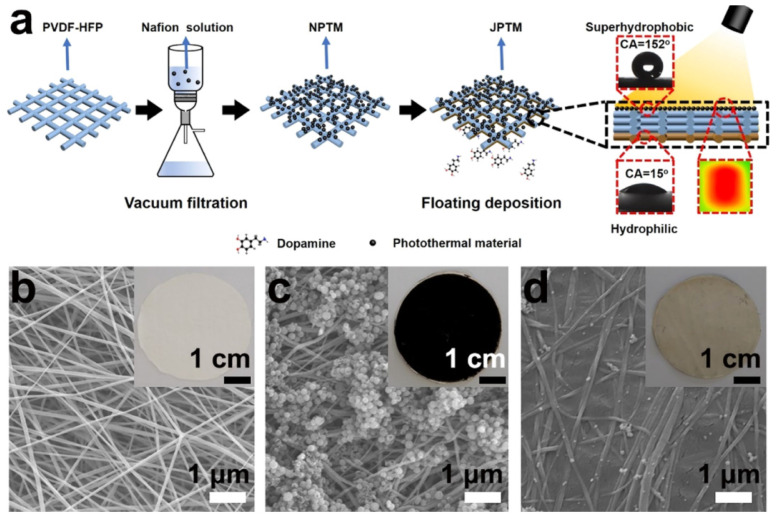
(**a**) Schematic diagram of the preparation of the JPTM; optical and SEM images of (**b**) bare PVDF-HFP membrane; (**c**) photothermal layer (feed side) of the JPTM; and (**d**) hydrophilic layer (output side) of the JPTM. Reprinted/adapted with permission from Ref. [88].

Immense research study on Janus membranes inspired Li and co-workers [88] to produce a novel Janus photothermal membrane (JPTM) that combines solar-capturing, permeation-enhancing, and antifouling properties in a single membrane for MD, as shown in Figure 13. The JPTM membrane was fabricated from a coating of the poly(vinylidene fluoride)-co-hexafluoropropylene (PVDF-HFP) nanofibrous membrane with polydopamine (PDA)-derived graphitic carbon spheres (GCSs). GCSs act as hydrophobic and photothermal functional layers on the feed side of the membrane. Meanwhile, on the bottom side of the membrane, polydopamine (PDA) was used as coating through in situ polymerization, which gives a superhydrophilic surface. They revealed that the superhydrophobic and photothermal layer of JPTM effectively increases the feed temperature through in situ heating, thus accelerating mass transport across the membrane in addition to improving its antifouling properties. The JPTM enhanced desalination when assembled into a solar-driven MD (SDMD) system. The distillation flux in the SDMD system showed 10 times increment than the conventional un-modified PVDF-HFP membrane at 1.29 kgm^−2^h^−1^, requiring only 1 kWm^−2^ solar simulator as an external heating source. On top of that, the as-prepares JPTM also showed a solar-photothermal (STT) conversion efficiency of up to 73%, which is among the highest reported for MD membranes to date. In addition, Wu et al. [89] have been fabricated by cPVA-PVDF/PMMA/GO Janus membranes by a layer-by-layer electrospinning, with varying hydrophilicity and hydrophobicity for oil–water separation. The Janus membranes generated by adding GO dispersion had dramatically better mechanical characteristics, pore size distribution, and contact angles, which additionally offered a superhydrophobic surface for the prepared Janus membranes. The hydrophilicity difference between the two sides is increased due to the presence of the superhydrophobic interface, and the effectiveness of the oil–water separation is increased. On the other hand, a more recent study was conducted by [90] have fabricated a Janus membrane that has a great potential for application of membrane distillation towards zero liquid discharge. In this work, they partially intruded pore channels of the fabricated Janus membrane (graphene oxide/poly (vinyl alcohol)/sulfosuccinic acid on polypropylene membrane) and the membrane exhibited superior antiwetting and antifouling performance for desalination and wastewater treatment in the DCMD distillation process. The stable water vapor fluxes and high salt rejections were also observed throughout the DCMD process with high water recovery (~80%). In addition, with 5 times leachate concentration, the Janus membrane were still exhibited superior robustness and wetting resistance.

### 3.2. Metal–Organic Frameworks

Over the last twenty years, metal–organic frameworks (MOFs) have captured great attention and achieved tremendous developments. MOFs are a class of porous hybrid materials with microporous crystalline structures formed by self-assembling metal ions/clusters and organic ligands. MOFs have an intensive capacity to combine inorganic metal centers with clustered centers of aluminum (Al), zirconium (Zr), and iron (Fe) with organic linkers by coordinate bonds without altering the framework [80] and exhibited a stable characteristic. They have been applied in various fields of separation due to their unique characteristics, such as tailorability, structural diversity and high specific surface area, making use of their high adsorption capacity [91].

A comprehensive review recently by Qadir et al. [92] stated that among the MOFs reported to date, Zn-based MOFs, particularly IRMOF-1, have been observed to be most moisture-sensitive due to their soft metal–oxygen coordination bonds, which are vulnerable to hydrolysis and lead to the disruption of the open framework structure. In addition, higher ligand basicity results in greater metal-ligand bond strength, affecting MOFs’ structural stability in aqueous media. For example, highly basic pyrazole (pKa 19.8) and imidazole (pKa 18.6) ligands exhibit higher chemical resistance to water than carboxylate-based MOFs. MOFs containing 6-coordinate (usually octahedral) metal centers were also more stable than those containing 4-coordinate (usually tetrahedral) metal centers, and metal centers or clusters with higher oxidation states could result in comparative higher stability towards reaction with water molecules.

**Figure 14:**
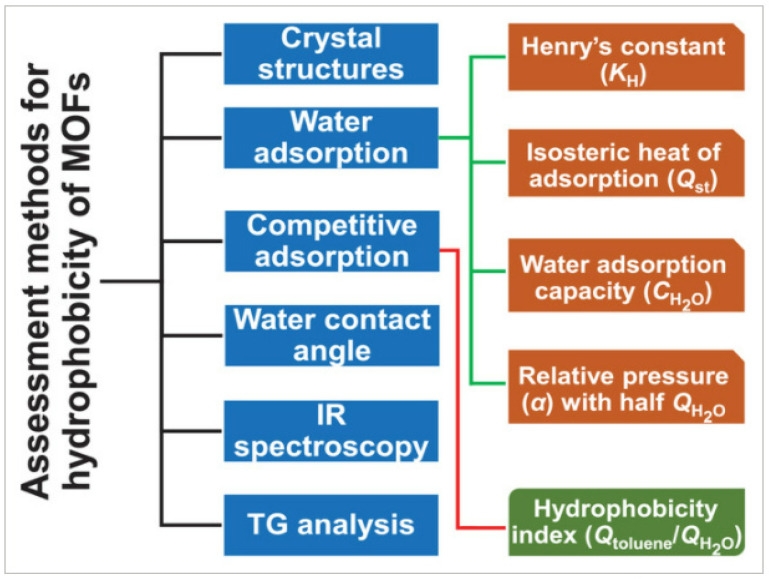
Methods and indicators for assessing the hydrophobicity of MOFs. Reprinted/adapted with permission from Ref. [94].

Nevertheless, prominently to date, MOFs-incorporated membranes have only been used for DCMD and VMD applications. In fact, limited works were also found for incorporating MOFs in nanofiber membranes or nanofiber as coating layers on membranes for MD applications. Xie et al. [93] reported that around tens of thousands of metal–organic frameworks (MOFs) have been developed in the past two decades. Still, only ≈100 have been demonstrated as porous and hydrophobic. Therefore, these would be the plausible reason why only limited works have been performed on MOFs-based nanofiber membranes for MD applications until now. However, a huge gap still needs to be explored in fine-tuning and optimizing the hydrophobicity of MOFs-nanofiber membranes. As shown in Figure 14, several methods and indicators are available to assess the hydrophobicity of MOFs. Nonetheless, organic ligands enable MOFs to form robust coordination interactions with polymeric membranes resulting in enhanced stability in the membrane [94]. Thus, upon the addition of MOFs, it still exhibited an increment in surface roughness of the membrane, thus subsequently enhancing the anti-wetting behavior [80].

In contrast, a recent report by Ni et al. [66] mentioned that the modified MOFs membranes could not reach omniphobicity and robust MD performance due to the MOFs’ hydrothermal instability. However, they also agreed that nanomaterial surface functionalization often provides membranes with re-entrant structures that assist a meta-stable of the Cassie–Baxter state, providing excellent wetting resistance for the membrane and surface roughness. These excellent features lead to the rise in hydrophobicity and evaporation areas. In the meantime, the functionalized particles can also stimulate the shear force, thus lessening the fouling potential by mitigating scale agglomeration.

Recent study performed by Yan et al. [91], they have fabricated a superhydrophobic poly(vinylidene fluoride) (PVDF) nanofibrous membranes with incorporation of MOF (iron 1,3,5-benzenetricarboxylate) via electrospinning on a non-woven substrate. They also applied a pre-treatment method called “solvent basing” to improve nanofibers’ attachment onto the substrate further. It was observed that the incorporation of 5 wt% of MOF in the membrane had successfully maintained a liquid entry pressure (LEP) of 82.73 kPa and increased the water contact angle of the membranes up to 138.06° ± 2.18° which is useful for DCMD. This is in agreement with the statement by Ray et al. [80] mentioned that an increase in the LEPw value can be linked to the reduced pore size, which in this case was due to the addition of MOF, thus as well increased hydrophobicity and wetting resistance of the membrane. On top of that, the membrane demonstrated 99.99% NaCl (35 g/L) rejection with 2.87 kg/m^2^ h of water vapor flux along the entire 5 h of the operating period. No leaching of MOF from the membranes was observed as inductively coupled mass spectrometry analysis indicated Fe^2+^ was found in the permeate.

A study by Efome et al. [95] successfully fabricated a novel membrane that produced potable water that surpasses drinking water standards in terms of conductivity through desalination by direct contact membrane distillation (DCMD). This smart membrane was assembled from a triple-layered nanofibrous membrane consisting of (1) a nanofiber layer electrospun from poly(vinylidene fluoride) (PVDF) blended with hydrophobic SiO_2_ NPs, (2) a nanofiber layer electrospun from polyacrylonitrile (PAN)/metal-organic frameworks (MOFs), and (3) a nanofiber layer electrospun from PVDF blended with hydrophilic SiO_2_ NPs. They found out the optimized loading of nanoparticles used in the membrane had exhibited the highest surface contact angle of 140.8° with LEPw, which was relatively low at 86.2 kPa. On top of that, the membrane also demonstrated an increment in the permeate flux of 4.40 kg/m^2^ h and permeate conductivity as low as 4 μS/cm during a 5 h operation. Several conclusions have been withdrawn from the findings, such as increased hydrophobic SiO_2_ NP loading had increased the WCA, present hydrophobicity properties and increased the pore size of the top PVDF layer. In addition, the incorporation of MOFs also increased the nanofiber diameter and pore size of the top and middle layer of PAN nanofiber, in addition to being very stable in water and suitable for use in producing pure water by desalination. These findings agree with Ray et al. [77] reported that mentioned MOFs revert the membrane hydrophobicity to hydrophilicity and improve the membranes’ wetting resistance and antifouling properties.

In addition, a new Janus composite membrane was developed by [96]. They modified the UiO-66-NH_2_ with polyvinyl alcohol (PVA) and immobilized the modified UiO-66-NH_2_ on the PTFE membrane through vacuum filtration. The finding from this study shows that the fabricated membranes could be a prospective alternative for treating wastewater containing oily substances and surfactants, where the modified Janus membrane demonstrated a high desalination rate of 99.99% within 24 h. The resulting membrane also produced a promising resistance to both membrane fouling and wetting of oils and surfactants with conductivity remaining lower than 10 μS/cm and the flux maintained at 21.3 L/(m^2^·h).

### 3.3. Metalloid and Metal Oxides-Based Nanoparticles

Metalloid and metal oxide nanoparticles (NPs) are extensively used to produce synergistic effects when combined with different types of membrane materials, enhance the properties of the membrane and generate superhydrophobic membranes surface for MD applications [80]. By nature, they own a hierarchal structure with multilevel roughness, thus improving the membrane’s hydrophobic character [95]. These rough surfaces naturally lead to the formation of air pockets, which allow for irregular contact between the water and the membrane surface, thus imposing the enhancement of the hydrophobic properties of the membrane as well as imparting a stable Cassie–Bexter regime with a high water contact angle (>150°) [97]. Among the metalloid and metal oxide NPs used for MD are Ag, Si, SiO_2_, TiO_2_, ZnO, and Al_2_O_3_. These NPs could be linked to the membranes via hydrogen or covalent bonds to ease the fabricated and tuneable multifunctional MD membranes [80].

**Figure 15:**
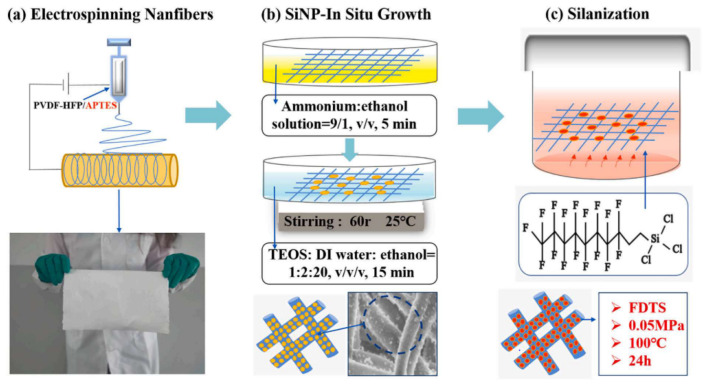
The detailed preparation process diagram of omniphobic membranes; (**a**) fabrication of porous nanofibers membrane using electrospinning approach, (**b**) In-situ growth of SiNPs on the PVDF-HFP/APTES substrates and (**c**) silanization process to obtain F-SiNPs/PVDF-HFP/APTES membrane. Reprinted/adapted with permission from Ref. [98].

As an emerging research interest, developing omniphobic membranes has attracted immense attention for MD applications. Xu et al. [98] have recently fabricated a novel omniphobic membrane for MD distillation fabricated by constructing a re-entrant microstructure via in situ silica nanoparticle (Si NPs) growth on an electrospun polyvinylidene fluoride-co-hexafluoropropylene (PVDF-HFP)/3-aminopropyl-triethoxysilane (APTES) nanofibrous membrane followed with subsequent hydrophobization process as shown Figure 15. APTES was introduced to provide nucleation sites for in situ SiNPs growth on the hydrophobic nanofibers and re-entrant structure construction. The as-prepared omniphobic membranes demonstrated superior omniphobicity properties with high contact angle values of 151.49°, 140.64°, 119.59°, and 107.5° to water, mineral oil, 4 mM sodium dodecyl sulfate (SDS) and ethanol, respectively. On top of that, the membranes exhibited robust scaling and acceptable wetting resistance, with salt rejection close to ~100%. A steady state permeates flux of 16.5 kg/m^2^ h and stable salt rejection efficiency was observed in a continuous 72 h operation, indicating outstanding anti-wetting and antifouling properties of omniphobic membranes.

**Figure 16:**
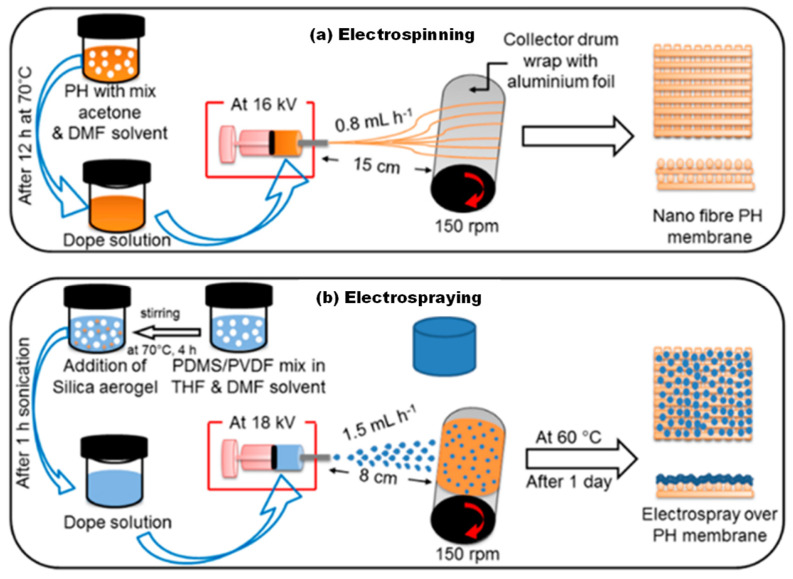
Illustration of dual−layer membrane fabrication by a combined electrospinning and electrospraying technique; (**a**) the supporting nanofiber layer was fabricated by electrospinning of dope solution and (**b**) the electrospraying process on membrane using dope solution. Reprinted/adapted with permission from Ref. [99].

Deka et al. [99] previously reported on the fabrication of a non-wettable membrane with re-entrant morphology and low surface energy with high roughness through electrospraying aerogel/PDMS/PVDF over a supporting scaffold layer of electrospun polyvinylidene fluoride-co-hexafluoropropylene (PVDF-HFP) as shown in Figure 16. Addition of hydrophobic silica-based aerogel in electrospun nanofiber membrane enhanced surface properties, water/ethanol/SDS contact angle, liquid entry pressure (LEP), pore size, porosity, surface roughness, and membrane performance, particularly for MD feeds containing low surface tension solvents. This dual-layer membrane achieved the highest superhydrophobicity (~170° water contact angle), liquid entry pressure (LEP) of 129.5 ± 3.4 kPa, short water droplet bouncing performance (11.6 ms), low surface energy (4.18 ± 0.27 mNm^−1^) and high surface roughness (Ra: 5.04 μm) with re-entrant structure upon present of 30% of aerogel (E-M3-A30) loading. A great non-wetting MD performance was also observed over a continuous 7 days operation of saline water (3.5% of NaCl), high antiwetting with harsh saline water containing 0.5 mM sodium dodecyl sulfate (SDS, 28.9 mNm^−1^), synthetic algal organic matter (AOM). However, they concluded that the omniphobicity of the as-prepared membrane has yet to be achieved as silica aerogel has oil absorbing. Therefore, they suggested that the introduction of NPs in future research works could possibly maintain the Cassie–Baxter state with organic solvents (superhydrophobicity with omniphobic properties) of the membrane.

In another work performed by Deka and co-workers [100], they recently successfully fabricated a dual−layer electrospun nanofiber membrane with superhydrophobic and omniphobic characteristics for the desalination of low-surface tension feed by MD. They functionalized the electrospun PVDF−HFP membrane using organosilane using 1H, 1H, 2H, 2H-perfluorooctyltriethoxysilane (FAS) to reduce the water sliding angle (SA) and grafting the membrane with fluorinated ZnO NPs (≤40 nm) to achieve regular and uniform re-entrant structures of the membrane. They found out that the optimized loading 25% *w*/*w* ZnO (ePFP-25Z), as-prepared membrane attained the highest contact angles for water (>161°), oil (131.5° ± 1.8°) and ethanol (131° ± 2.9°), had high surface roughness (3.26 μm) and lowest surface energy (0.75 ± 0.43 mNm^−1^). In addition, the PFP−25Z membrane also retained a stable membrane distillate flux and salt rejection (>99.9%) after 80 h operation. This might plausibly be due to the superior properties of ZnO NPs, including desirable physical appearance, high surface-to-volume ratio, enhanced thermal resistance, and superior bactericidal, environmentally friendly and economical characteristics compared to SiO_2_ and TiO_2_ [101,102].

**Figure 17:**
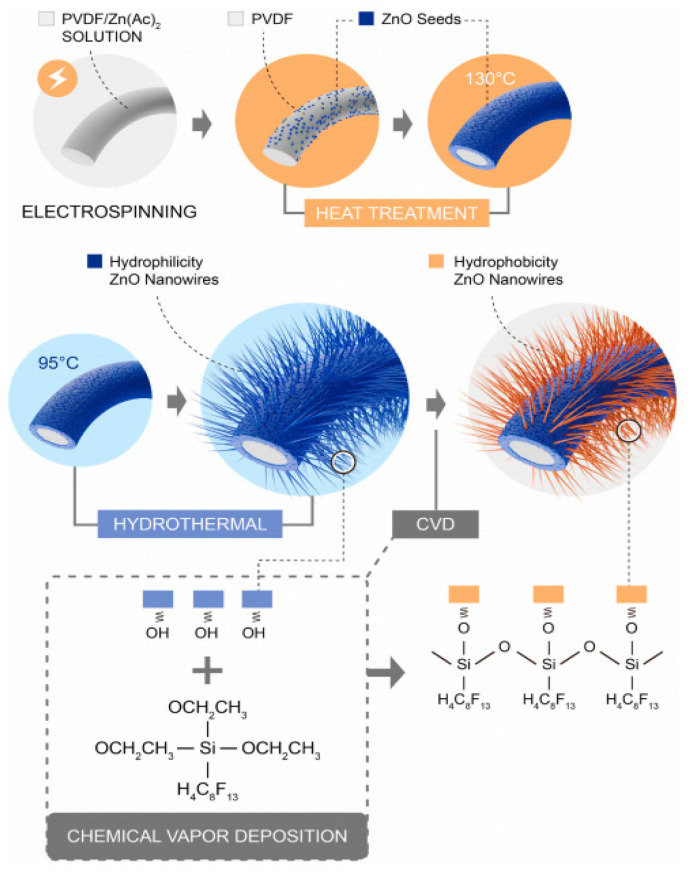
Schematic of the fabrication of P–ZnO@PVDF membrane. Reprinted/adapted with permission from Ref. [44].

The great performance of the ZnO-based nanofiber membrane for MD was also reported by Pane et al. [44], where they revealed that the modified nanofiber membrane demonstrated enhanced anti-wetting and anti-scaling properties. This was attributed to its superhydrophobicity, increased heterogeneous nucleation barrier, and reduced contact area and contact time between fluid and the membrane surface. This study fabricated a hybrid composite nanofiber membrane of ZnO nanowires/PVDF nanofiber membrane (P–ZnO@PVDF) based on the hydrothermal method and chemical vapor deposition (CVD), as shown in Figure 17. The adhesion between ZnO nanowires and PVDF nanofiber membrane was enhanced by mimicking grass growth. Upon addition of ZnO nanowires, the P–ZnO@PVDF membrane exhibited narrow pore size distribution, robust structure, high porosity, superhydrophobicity, anti-wetting and anti-scaling properties due to the slip and Cassie Baxter state. Findings from the experiment demonstrated high salt rejection at 99.9% and a stable permeation flux of 15.7 L m^−2^ h^−1^ after 60 h operation, and it was recommended as the practical application of long-term operation MD.

On the other hand, Huang and co-workers [103] have proposed a novel method of suppressing the temperature polarization of MD by heating the hydrophobic membrane surface through the photothermal effects of antimony-doped tin oxide (ATO) NPs (average size: 20–40 nm). The fabricated hybrid polyvinylidene fluoride (PVDF)/ATO nanofiber membranes had good photothermal heating generated by infrared-induced thermal to raise the membrane’s surface temperature significantly. Based on the study, they found that the thermal radiation energy of ATO raised about 13 °C, thus effectively a reduction in temperature polarization. The assistance of infrared radiation elevated the permeate flux of (PVDF)/ATO nanofiber membranes, wherein the highest loading of ATO had significantly increased permeate flux from about 8.0 L m^−2^ h^−1^ to 27.0 L m^−2^ h^−1^.

Ren et al. [62] performed a novel super-hydrophobization method to surface modify nanofibrous PVDF membranes via the incorporation of titanium dioxide (TiO_2_) and 1H, 1H, 2H, 2H-perfluorododecyl trichlorosilane (FTCS) for desalination by DCMD system. As prepared, TiO_2_-FTCS modified PVDF nanofiber membranes possessed high roughness and hydrophobicity (157.1°) with a wetting resistance of 158 kPa, surface porosity of 57%, high mean roughness of 4.63 μm, well-distributed pore size approximately 0.81 μm and modest membrane thickness around 55 μm. They found that the membranes exhibited high flux and stable desalination performances with achieved permeate flux of 73.4 LMH (L/m^2^ h) and 99.99% salt rejection during the short-term DCMD process. Meanwhile, the 40.5 LMH permeate flux with 99.98% salt rejection was achieved for the long-term DCMD process. These great findings significantly surpassed the performance of commercial PVDF membranes and unmodified PVDF nanofiber membranes, indicating the potential for TiO_2_-FTCS-modified PVDF nanofiber membranes in DCMD applications. Table 1 summarizes the recent state-of-the-art nanomaterials modified nanofiber membranes for MD applications.

## 4. Future Way Forward and Conclusions

The main goal of this mini-review is to provide insights on fouling issues in the membrane distillation process, particularly using electrospun nanofibrous membranes and nanofiber membranes and the progress performed to improve and optimize its performance in water and wastewater treatment. Although a myriad of research studies have been conducted to enhance the fouling resistance of membranes in membrane distillation for water and wastewater treatment, there remains a huge gap for the MD process to be efficiently operated at a larger scale of operation. The introduction of superhydrophobic, omniphobic, and Janus membranes to modify the properties of nanofiber membranes reduces the fouling tendency, increases the porosity of the membrane, and improves the mechanical and chemical stability, thereby ensuring the production of a superior MD membrane class, according to extensive literature studies. Improvement of these properties enables the fabrication of robust anti-wetting surfaces for long-term MD operations. Altering the surface-wetting properties of these MD membranes by incorporating the membrane with nanomaterials has shown remarkable achievement in the high performance of MD membranes. However, several problems, such as scalability, high fabrication costs, difficult methods of designing and the hazardous nature of the incorporated materials in the membrane matrix, are commonly observed. Therefore, to establish the work performed in small-scale laboratories to huge-scale industries, future research is anticipated to explore the discovery of more viable, feasible and successful solutions for MC applications. Thus, intensive collaborative studies between university and industry practitioners are extremely desirable as these novel membranes must be commercialized globally to extend the utilization of MD in large-scale water treatment facilities.

## Figures and Tables

**Table 1:** Recent state-of-the-art nanomaterials modified nanofiber membranes for MD applications.

Nanofiber-Based Membrane	Nano- Materials	MD Type	Category	Contact Angle	Flux (kg/m^2^ h)	Salt Rejection	Ref.
PVDF	CNT	VMD	Carbon	159.3°	28.4	>99.99%	[53]
PVDF	MWCNT	DCMD	Carbon	142.0°	74.7	99.995%	[85]
PVDF	GO	DCMD	Carbon	119.0°	28 L	>99.90%	[86]
PVDF-HFP	GO	AGMD	Carbon	162.0°	21.1	99.99%	[87]
PP	GO	DCMD	Carbon	120.0°	3.4	99.60%	[90]
PVDF-HFP	Carbon spheres	SDMD	Carbon	152.0°	1.29	99.99%	[88]
PVDF	F300	DCMD	MOF	138.06°	2.87	99.99%	[91]
PAN/ PVDF	MOF-808	DCMD	MOF	140.0°	4.4	99.97%	[95]
PTFE	UiO-66-NH_2_	DCMD	MOF	54.0°	21.3	99.99%	[96]
PVDF-HFP/APTES	Si	DCMD	Metalloid	154.9°	16.5	~100.00%	[98]
PVDF-HFP	Si	DCMD	Metalloid	170.0°	34.6	99.90%	[99]
PVDF-HFP-FAS	ZnO	DCMD	Metal oxide	>161.0°	22.7	>99.90%	[100]
PVDF	ZnO	DCMD	Metal oxide	150.0°	15.7	99.90%	[44]
PVDF	ATO	VMD	Metal oxide	125.5°	27.0	99.00%	[103]
PVDF	TiO_2_	DCMD	Metal oxide	157.1°	73.4	99.99%	[62]

## Data Availability

The reference journals used and/or reviewed for the current study are available from the corresponding author on reasonable request.
